# Bioprocessing of mixed oil palm by-products with single and consortium white-rot fungi: Effects on nutritional quality, in vitro rumen fermentation, methane production, and rumen microbial populations

**DOI:** 10.14202/vetworld.2026.3774-3792

**Published:** 2026-08-27

**Authors:** Fenda Alvionita Fhonna, Anuraga Jayanegara, Sitti Wajizah, Anjas Asmara Samsudin, Samadi Samadi

**Affiliations:** 1 Doctoral Program of Agricultural Science, Postgraduate School, Universitas Syiah Kuala, Banda Aceh 23111, Indonesia.; 2 Department of Nutrition and Feed Technology, Faculty of Animal Science, IPB University, Bogor 16680, Indonesia.; 3 Department of Animal Husbandry, Faculty of Agriculture, Universitas Syiah Kuala, Banda Aceh 23111, Indonesia.; 4 Research Center for Innovation and Feed Technology, Universitas Syiah Kuala, Banda Aceh 23111, Indonesia.; 5 Institute of Tropical Agriculture and Food Security, Universiti Putra Malaysia, Selangor 43400, Malaysia.

**Keywords:** biological pretreatment, fungal consortium, lignocellulosic biomass, methane mitigation, oil palm by-products, Phanerochaete chrysosporium, Trametes versicolor, white-rot fungi

## Abstract

**Background and Aim:**

Oil palm by-products are abundant lignocellulosic resources with considerable potential as sustainable ruminant feed but are constrained by poor digestibility and limited rumen fermentability. White-rot fungi (WRF) produce ligninolytic enzymes capable of modifying lignocellulosic biomass, whereas fungal consortia may enhance substrate bioconversion through complementary enzymatic activities. This study evaluated the effects of single and consortium WRF on the nutritional quality, *in vitro* rumen fermentation, digestibility, methane (CH_4_) production, and rumen microbial populations of mixed oil palm by-products.

**Materials and Methods:**

A substrate comprising oil palm fronds, palm kernel cake, and oil palm decanter cake was subjected to 28-day solid-state fermentation using 6% (w/w) inoculum. Five treatments were evaluated: Unfermented control, fermented control without fungal inoculation, *Phanerochaete* *chrysosporium*, *Trametes versicolor*, and their consortium, each with five biological replicates. Nutrient composition was determined after fermentation. *In vitro* gas production kinetics, rumen fermentation characteristics, CH_4_ production, digestibility, and metabolizable energy (ME) were evaluated over 72 h using buffered rumen fluid. Rumen microbial populations were quantified by quantitative polymerase chain reaction.

**Results:**

Fermentation significantly increased crude protein (CP) by 24.8%–34.8% while decreasing ether extract, hemicellulose, and non-fiber carbohydrates (p < 0.05). Conversely, relative lignin concentration and structural fiber fractions increased, indicating preferential carbohydrate utilization during fungal growth. All fermented treatments reduced cumulative gas production, total volatile fatty acids, CH_4_ production, digestibility, and ME compared with the unfermented control (p < 0.05). CH_4_ production declined by 26.6%–33.6%, although this reduction was accompanied by lower fermentation efficiency rather than improved feed utilization. Ruminal pH and ammonia nitrogen remained within acceptable ranges and were unaffected by fungal inoculation. Total bacterial abundance differed among treatments, whereas the populations of *Fibrobacter succinogenes* and *Ruminococcus albus* remained unchanged. The consortium treatment did not provide additional benefits over single-fungus fermentations for nutrient utilization or rumen fermentation responses.

**Conclusion:**

Bioprocessing mixed oil palm by-products with single or consortium WRF altered substrate composition by increasing CP while reducing CH_4_ production. However, these changes were accompanied by reduced rumen fermentability, digestibility, and energy availability, indicating that the tested fermentation conditions did not improve the overall feeding value of the mixed substrate. Consortium fermentation offered no clear advantage over single fungal cultures, highlighting the need to optimize fungal species combinations and fermentation conditions before practical application as a biological pretreatment for ruminant feeds.

## INTRODUCTION

Oil palm by-products have attracted considerable attention as alternative feed resources because of their abundant availability in major palm oil-producing countries. Indonesia and Malaysia collectively account for approximately 82% of global palm oil production [1] and generate substantial quantities of lignocellulosic by-products, including oil palm fronds (OPF), palm kernel cake (PKC), and oil palm decanter cake (OPDC) [2, 3]. In 2023, annual OPF production in Indonesia and Malaysia was estimated at approximately 156.5 and 59.3 million tons, respectively [4]. These by-products possess complementary nutritional characteristics, making their combined utilization a practical strategy for formulating balanced ruminant diets from locally available oil palm residues. OPF serves as an abundant fibrous resource [5], whereas PKC and OPDC provide relatively higher concentrations of protein and energy [6], thereby partially compensating for the nutritional limitations of OPF. This integrated utilization strategy is particularly relevant in Indonesia and Malaysia, where these by-products are generated simultaneously throughout the oil palm production chain. However, their extensive lignocellulosic structure substantially limits their utilization as ruminant feed. A recent meta-analysis demonstrated that lignification of PKC and OPF is a major constraint that reduces nutritional value, rumen fermentation efficiency, degradability, and animal performance, emphasizing the need for effective pretreatment strategies to improve their feeding value [7, 8].

Various physical, chemical, and biological pretreatment methods have been investigated to enhance the nutritional quality of oil palm by-products. However, each approach has inherent limitations, including incomplete lignin degradation, high operational costs, potential toxicological and environmental concerns, and prolonged processing times [9, 10]. Among these methods, biological pretreatment has emerged as a promising alternative because it improves substrate quality through a safer, environmentally friendly, and sustainable process [11]. In particular, white-rot fungi (WRF) have attracted considerable attention for their exceptional ligninolytic capabilities and their ability to selectively modify lignocellulosic biomass.

WRF are ligninolytic fungi capable of degrading lignin through extracellular oxidative enzymes [12]. Different WRF species possess distinct enzymatic systems that influence the efficiency of lignocellulose degradation. Through the production of lignin peroxidase (LiP), manganese peroxidase (MnP), and laccase (Lac), WRF depolymerize lignin, thereby increasing the accessibility of structural carbohydrates to rumen microorganisms [11, 13]. Among these fungi, *Phanerochaete* *chrysosporium* is characterized by strong LiP and MnP activities, whereas *Trametes versicolor* exhibits high Lac activity, suggesting complementary ligninolytic mechanisms and the potential for synergistic interactions when both species are applied as a consortium [14]. Compared with monocultures, WRF consortia may exhibit greater enzymatic diversity and activity, thereby enhancing delignification and improving *in vitro* digestibility and fermentability of lignocellulosic biomass [14, 15]. Consequently, combining these fungi represents a promising biological pretreatment strategy for improving the nutritional value of mixed oil palm by-products.

Although the application of WRF for upgrading lignocellulosic feed resources has been extensively investigated, previous studies have predominantly evaluated individual fungal species using single oil palm by-products, such as OPF, PKC, or OPDC [16–18]. Consequently, information on the comparative performance of single- and consortium-WRF models for mixed oil palm by-products remains limited. Moreover, no previous study has comprehensively evaluated the effects of single and consortium fermentations under identical experimental conditions by simultaneously assessing changes in nutrient composition, *in vitro* gas production kinetics, rumen fermentation characteristics, digestibility, methane (CH₄) production, and rumen microbial populations. This lack of integrated evidence limits the understanding of whether consortium WRF provides synergistic advantages over single fungal cultures for biological pretreatment of mixed oil palm by-products intended for ruminant feeding.

Therefore, this study aimed to evaluate the effects of single and consortium WRF fermentation on the nutritional quality and *in vitro* rumen responses of a mixed substrate comprising OPF, PKC, and OPDC. The evaluation included nutrient composition, cumulative gas production and gas production kinetics, rumen fermentation characteristics, digestibility, CH₄ production, and rumen microbial populations quantified using quantitative polymerase chain reaction (qPCR). It was hypothesized that consortium WRF would exhibit complementary ligninolytic activity and improve substrate utilization more effectively than single fungal cultures. The findings are expected to provide novel insights into the biological valorization of mixed oil palm by-products and contribute to the development of sustainable ruminant feeding strategies within a circular bioeconomy framework.

## MATERIALS AND METHODS

### Ethical approval

Ethical approval was not required because no live animals were used in this study. Rumen fluid was collected from cattle after slaughter at a licensed abattoir as a by-product of routine slaughterhouse processing. Therefore, no experimental procedures were performed on live animals.

### Study period and location

The study was conducted from July to October 2025 at the Nutrition, Technology, and Forage Science Laboratory, Faculty of Agriculture, Universitas Syiah Kuala, Banda Aceh, Aceh, Indonesia, and the Nutrition Laboratory, Faculty of Agriculture, Universiti Putra Malaysia, Serdang, Selangor, Malaysia.

### Experimental design and treatments

The experiment was conducted using a completely randomized design to evaluate the effects of single and consortium WRF fermentation on the nutrient composition, fermentability, *in vitro* digestibility, and rumen microbial populations of mixed oil palm by-products. The experimental substrate consisted of OPF, PKC, and OPDC formulated on a dry matter (DM) basis. Five treatments were evaluated: An unfermented control (CON), a fermentation control without fungal inoculation (FCON), fermentation with *P. chrysosporium* (FPC), fermentation with *T. versicolor* (FTV), and fermentation with a consortium of *P. chrysosporium* and *T. versicolor* (FPCTV). Fermentation was conducted for 28 days at 29.35 ± 0.37°C and a relative humidity of 66.57 ± 0.40%. Each treatment consisted of five independent biological replicates.

### Preparation of oil palm by-products

OPF was obtained from Sei Putih, Galang, Deli Serdang Regency, North Sumatra, Indonesia, whereas PKC and OPDC were obtained from Bandar Sinembah, Binjai, North Sumatra, Indonesia. The OPF was chopped into pieces measuring 1–3 cm before drying. The PKC was passed through a 2-mm mesh sieve to remove coarse particles before formulation. The OPDC was oven-dried at 60°C until a constant weight was achieved and then ground to pass through a 2-mm sieve.

The DM content of each ingredient was determined and used as the basis for substrate formulation. The basal substrate contained 50% OPF, 25% PKC, and 25% OPDC on a DM basis. This formulation was selected from several candidate mixtures because it had the highest crude protein (CP) concentration and the lowest crude fiber content. The chemical compositions of OPF, PKC, OPDC, and the experimental mixture are presented in Table 1.

**Table 1:** Chemical composition of the experimental substrates.

**Chemical composition (% DM)**	**OPF**	**PKC**	**OPDC**	**Mixed oil palm by-products**
Dry matter	80.29	93.75	92.71	91.08
Ash	3.73	6.51	17.21	8.92
Ether extract	0.14	6.04	8.53	2.50
Crude protein	3.52	18.35	16.85	10.17
NDF	87.96	75.37	60.02	70.83
ADF	72.16	44.84	50.16	51.07
Cellulose	57.33	30.70	17.64	38.27
Hemicellulose	15.80	30.53	9.86	19.76
Lignin	14.83	14.14	32.52	12.80
Gross energy (MJ/kg DM)	15.15	19.14	21.19	18.06

DM = Dry matter, OPF = Oil palm fronds, PKC = Palm kernel cake, OPDC = Oil palm decanter cake, NDF = Neutral detergent fiber, ADF = Acid detergent fiber.

### WRF and inoculum preparation

*P. chrysosporium* InaCC F206 and *T. versicolor* InaCC F200 were obtained from the Indonesia Culture Collection (InaCC, Cibinong, Indonesia) and rejuvenated on potato dextrose agar. The inoculated cultures were incubated at room temperature (27–28°C) for 7 days before inoculum preparation.

Fungal spawn was prepared using ground maize as the carrier substrate. The maize was washed, soaked overnight, drained, transferred into polypropylene bags at 250 g/bag, and sterilized in an autoclave at 121°C for 30 min. After cooling to room temperature, each bag was aseptically inoculated with ten 1-cm² agar plugs containing actively growing fungal mycelia. The bags were manually shaken to ensure uniform distribution of the mycelia throughout the maize. The inoculated maize was incubated at room temperature (27–28°C) for 2 weeks until complete mycelial colonization was observed. The fully colonized spawn was stored at 4°C to inhibit further fungal growth until its subsequent use in fermentation [19].

### Solid-state fermentation (SSF) process

SSF was conducted using a modified method described by Yan *et al.* [20]. The substrate mixture was supplemented with 6% corn bran and 4% molasses on a DM basis to provide additional energy and nutrients for fungal growth. Sterile water was added to adjust the substrate moisture content to 60% (DM basis), and all ingredients were thoroughly mixed and manually homogenized.

For FPC and FTV, the substrate was inoculated with colonized maize spawn at 6% (w/w). For FPCTV, the substrate was inoculated with 3% (w/w) spawn from each fungus, yielding a total inoculum concentration of 6% (w/w). FCON received no fungal inoculum, whereas CON underwent the same substrate preparation procedure but was not incubated. Each treatment consisted of five independently prepared fermentation bags.

After homogenization, 900 g DM of substrate was packed into each heat-resistant polypropylene bag. Aerobic conditions were maintained by perforating the bags to permit air exchange during incubation. The substrates were incubated for 28 days under ambient laboratory conditions at 29.35 ± 0.37°C and 66.57 ± 0.40% relative humidity (mean ± standard deviation; n = 28). The incubation period was selected based on previous studies of lignocellulosic substrate degradation by *P. chrysosporium* and *T. versicolor* [21–23].

After incubation, fermentation was terminated by oven-drying the substrates at 60°C for 72 h to halt biological activity and stabilize the samples. The dried substrates were ground to pass through a 1-mm sieve and stored in sealed bags until subsequent chemical and *in vitro* analyses.

### Chemical composition analysis

The proximate compositions of the experimental ingredients and samples were determined according to the official methods of the Association of Official Analytical Chemists [24]. The analyses included moisture, ash, CP, and ether extract (EE). Gross energy was determined using an IKA® C 3000 bomb calorimeter (IKA-Werke GmbH & Co. KG, Staufen, Germany).

Neutral detergent fiber (NDF), acid detergent fiber (ADF), and acid detergent lignin (ADL) were determined according to Van Soest *et al.* [25]. Hemicellulose concentration was calculated as the difference between NDF and ADF, whereas cellulose concentration was calculated as the difference between ADF and ADL. ADL was used as an estimate of lignin concentration. The NDF residues were ashed at 550°C for 3 h, and ash-corrected NDF (NDFom) was calculated by subtracting the residual ash from the NDF residue.

### *In vitro* rumen fermentation

**Rumen fluid collection:** Rumen fluid was collected in the morning from three freshly slaughtered beef cattle at the Shah Alam Abattoir, Selangor, Malaysia. Information on the feeding regimen prior to slaughter was unavailable; however, all donor animals originated from the same facility. Immediately after collection, the rumen fluid was transferred into pre-warmed vacuum flasks and continuously flushed with carbon dioxide (CO₂) to maintain anaerobic conditions. Equal volumes of rumen fluid from each donor animal were pooled to minimize biological variation and obtain a representative rumen inoculum. The pooled rumen fluid was filtered through three layers of cheesecloth to remove coarse feed particles, then transferred into Erlenmeyer flasks maintained in a water bath at 39°C and mixed with a standard *in vitro* buffer solution at a 1:2 (v/v) ratio while continuously flushing with CO₂ to maintain anaerobiosis.

### Buffer medium preparation

The rumen buffer medium was prepared by mixing 620 mL distilled water, 0.15 mL micro-mineral solution, 310 mL buffer solution, 310 mL macro-mineral solution, 2 mL resazurin solution, and 62 mL reducing solution. The mixture was continuously stirred and flushed with CO₂ until the resazurin indicator turned pale pink or colorless, indicating that a reducing anaerobic environment had been established.

**Incubation procedure:** *In vitro* rumen fermentation was performed as described by Menke and Steingass [26]. Approximately 0.2 g of each oven-dried sample (60°C) was weighed into a 100-mL calibrated glass syringe (Fortuna GmbH, Wertheim, Germany). A standard alfalfa hay sample of equivalent weight was included as the reference substrate, whereas blank syringes containing only buffered rumen inoculum without substrate were prepared to correct for background gas production. Buffered rumen medium (30 mL) was dispensed into each syringe containing the treatment substrate, reference substrate, or blank. Each biological replicate (fermentation bag) was incubated in duplicate using independent syringes, whereas the reference substrate and blank were incubated in triplicate.

**In vitro gas production:** Incubation was continued for 72 h, and cumulative gas production was recorded at 2, 4, 6, 8, 10, 12, 14, 16, 18, 20, 22, 24, 30, 36, 48, and 72 h. Net gas production was calculated by subtracting the gas volume produced in the blank syringes from the corresponding treatment values. Gas production data were fitted to the nonlinear model of Ørskov and McDonald [27] using the Nonlinear Regression procedure in IBM SPSS Statistics version 27.0 (IBM Corp., Armonk, NY, USA) to estimate gas production kinetic parameters. The goodness-of-fit of each model was evaluated using the coefficient of determination (R²). Cumulative gas production was expressed as mL/200 mg DM after correction for sample weight and DM content.

At the end of incubation, rumen fluid was collected to determine ruminal pH, *in vitro* dry matter digestibility (IVDMD), ammonia nitrogen (NH₃-N) concentration, total and individual volatile fatty acid (VFA) concentrations, and rumen microbial populations using qPCR.

**In vitro digestibility and metabolizable energy (ME):** Following incubation, fermentation residues were recovered by vacuum filtration through pre-weighed sintered-glass crucibles. The residues were rinsed with distilled water and oven-dried at 105°C until a constant weight was obtained. IVDMD was calculated according to Menke and Steingass [26] using the following equation:

IVDMD (%) = 100 × [(Initial sample DM − Residual DM − Blank)/Initial sample DM]

ME and *in vitro* organic matter digestibility (IVOMD) were estimated according to Menke and Steingass [26] using the following equations:

ME (MJ/kg DM) = 1.24 + 0.146(GP) + 0.0070(CP) + 0.0224(EE)

IVOMD (%) = 15.38 + 0.8453(GP) + 0.0595(CP) + 0.0675(Ash)

where GP is the gas volume (mL) produced from 200 mg DM after 24 h of incubation, CP is crude protein (g/kg DM), EE is ether extract (g/kg DM), and ASH is crude ash (g/kg DM).

### Rumen fermentation characteristics

**Ruminal pH:** Ruminal pH was measured after 72 h of incubation using a pH meter (Mettler-Toledo Ltd., Leicester, UK). Approximately 10 mL of rumen fluid was collected from each syringe and stored for subsequent determination of NH₃-N concentration, VFA profile, and rumen microbial populations.

**NH₃-N concentration:** The NH₃-N concentration was determined using the indophenol method [28], which is based on the formation of a blue-colored indophenol complex measured spectrophotometrically. Buffer solution and 1 N sodium hydroxide were used to prepare the phenol-nitroprusside and hypochlorite reagents, respectively. All reagents were stored in the dark until use.

Rumen fluid (2.5 mL) was mixed with 2.5 mL distilled water and 2 mL phenol-nitroprusside solution and vortexed thoroughly. Subsequently, 3 mL hypochlorite reagent was added, and the reaction mixture was homogenized and incubated at room temperature for 1 h. Development of a blue color indicated the presence of ammonia. Absorbance was measured at 635 nm with a GENESYS™ 20 spectrophotometer (Thermo Scientific™, Waltham, MA, USA), calibrated with a reagent blank. Standard ammonium chloride solutions (0.01, 0.1, 0.5, and 1 mM) were processed identically to generate a standard curve, and NH₃-N concentrations were calculated from the corresponding linear regression equation.

**VFA concentration:** The VFA profile was determined by gas chromatography, as described by Nur Atikah *et al.* [29]. Frozen rumen fluid samples were thawed at room temperature before analysis. An aliquot of 2 mL rumen fluid was transferred into a microcentrifuge tube and acidified with 200 μL of a 3:1 (v/v) mixture of 25% metaphosphoric acid and 5% formic acid. The mixture was incubated at room temperature for 30 min and centrifuged at 3,000 × *g* for 10 min at 24°C. The supernatant was filtered through a 0.45-μm syringe filter and transferred into a 1.5-mL screw-cap glass vial (Supelco, Sigma-Aldrich, St. Louis, MO, USA). An internal standard, 4-methyl-*N*-valeric acid (Sigma-Aldrich), was added to the filtrate.

Individual VFAs, including acetate, propionate, iso-butyrate, butyrate, iso-valerate, and valerate, were separated and quantified using a 6890N Network GC System (Agilent Technologies, Santa Clara, CA, USA) equipped with a flame ionization detector and a 15-m fused silica capillary column (Quadrex 007 Series, Quadrex Corp., New Haven, CT, USA; 0.250-mm internal diameter and 0.25-μm film thickness). Nitrogen was used as the carrier gas at a flow rate of 60 mL/min. The column temperature was maintained at 200°C, whereas the injector and detector temperatures were set at 230°C.

Peak identification was performed using a Volatile Free Acid Mix (Sigma-Aldrich) containing 20 mM acetate and 10 mM each of propionate, butyrate, and 4-methylvalerate as the external standard. Individual VFA concentrations were calculated using a single-point calibration method based on the internal and external standards.

**CH₄ production:** CH₄ production was estimated indirectly from the molar proportions of VFA produced during *in vitro* rumen fermentation according to Moss *et al.* [30] using the following equation:

CH₄ (mmol/L) = 0.45(C₂) − 0.275(C₃) + 0.40(C₄)

Adjusted CH₄ production was subsequently calculated according to Jayanegara *et al.* [31]:

Adjusted CH₄ = CH₄ × 100/Hrec

Hydrogen recovery (Hrec) was calculated according to Demeyer and Van Nevel [32] as follows:

Hrec (%) = (2Hp/2Hu) × 100

where 2Hp represents the total hydrogen produced and 2Hu represents the total hydrogen utilized. These values were calculated from the molar concentrations of acetate (C₂), propionate (C₃), butyrate (C₄), iso-valerate (isoC₅), valerate (C₅), and CH₄ using the following equations:

2Hu = 2(C₃) + 2(C₄) + 4(CH₄) + C₅

2Hp = 2(C₂) + C₃ + 4(C₄) + 2(isoC₅) + 2(C₅)

**Quantitative analysis of rumen microbes by qPCR:** Rumen microbial populations were quantified after 72 h of incubation. Microbial DNA was extracted using the PrimeWay Stool DNA Extraction Kit (KIT-9070; 1st BASE, Selangor, Malaysia) according to the manufacturer's instructions. DNA concentration and purity were determined using a µDrop™ Duo Plate spectrophotometer (Thermo Scientific™, Waltham, MA, USA). Samples with sufficient DNA quantity and quality were selected for qPCR analysis, resulting in four biological replicates per treatment. Each biological replicate was analyzed in duplicate.

Quantitative PCR was performed using SYBR Green chemistry on an Azure Cielo™ Real-Time PCR System (Azure Biosystems, Dublin, CA, USA) with optical reaction plates compatible with fluorescence detection. The target microbial populations and primer sequences are presented in Table 2 [33–36]. Each 20-µL reaction mixture consisted of 10 µL THUNDERBIRD™ Next SYBR™ qPCR Mix (TOYOBO Co., Ltd., Osaka, Japan), 1 µL each of the forward and reverse primers, 2 µL template DNA, and RNase-free distilled water to a final volume of 20 µL. Reaction mixtures were dispensed into 0.2-mL RNase/DNase-free thin-walled PCR 8-strip tubes with transparent caps (Servicebio, Wuhan, China).

Each qPCR assay included no-template controls (NTC) to monitor contamination and positive controls at multiple concentrations to verify assay performance. Thermal cycling consisted of an initial enzyme activation step at 95°C for 30 s, followed by 40 cycles of denaturation at 95°C for 5 s, annealing at 60°C for most primer pairs and 53°C for *Fibrobacter succinogenes* [34] for 10 s, and extension at 72°C for 20 s. Amplicon specificity was confirmed by melting curve analysis with a gradual temperature increase from 60°C to 90°C.

Microbial populations were quantified using the absolute quantification method. Standard curves for each microbial target were generated from 10-fold serial dilutions of purified PCR products obtained from pure cultures of the corresponding microorganisms. Microbial abundance was calculated from the quantification cycle (Cq) values using the respective standard curves. The standard curves exhibited a mean amplification efficiency of 106.8% with an R² of 0.9995.

**Table 2:** Primers used for qPCR analysis.

**Target microorganism**	**Primer**	**Sequence (5′–3′)**	**Reference**
Total bacteria	F	CGGCAACGAGCGCAACCC	[33]
	R	CCATTGTAGCACGTGTGTAGCC	
*Fibrobacter* * succinogenes*	F	GGTATGGGATGAGCTTGC	[34]
	R	GCCTGCCCCTGAACTATC	
*Ruminococcus* * albus*	F	TGTTAACAGAGGGAAGCAAAGCA	[35]
	R	TGCAGCCTACAATCCGAACTAA	
*Ruminococcus* *flavefaciens*	F	TGGCGGACGGGTGAGTAAAGCT	[35]
	R	TTACCATCCGTTTCCAGA	
Total protozoa	F	GCTTTCGWTGGTAGTGTATT	[36]
	R	CTTGCCCTCYAATCGTWCT	

qPCR = Quantitative polymerase chain reaction, F = Forward primer, R = Reverse primer.

### Statistical analysis

Data were analyzed using IBM SPSS Statistics version 27.0 (IBM Corp., Armonk, NY, USA). One-way analysis of variance (ANOVA) was used to compare treatment means, followed by Duncan's multiple range test for pairwise comparisons when significant differences were detected (p < 0.05).

Before ANOVA, normality was assessed using the Shapiro-Wilk test, and homogeneity of variances was evaluated using Levene's test. Variables that violated the homogeneity assumption, including ash, ADF, lignin, non-fiber carbohydrates (NFC), gross energy, NH₃-N, valerate, and gas production at 2 and 3 h, were analyzed using Welch's ANOVA followed by the Games-Howell multiple comparison test (p < 0.05).

Relationships between nutrient composition and *in vitro* fermentation characteristics were evaluated using Pearson correlation analysis. Data are presented as mean ± standard error (SE). The fermentation bag served as the experimental unit, with five independent biological replicates per treatment (n = 5).

## RESULTS

### Nutrient composition

Fermentation with WRF, either as single inocula (FPC and FTV) or as a consortium (FPCTV), significantly affected the nutrient composition of the mixed oil palm by-product substrate (Table 3). Fermentation significantly reduced DM content compared with CON (p < 0.001). CP, ash, and gross energy contents were significantly higher in FCON, FPC, FTV, and FPCTV than in CON (p < 0.01). In contrast, EE, hemicellulose, and NFC contents were significantly lower in all fermented treatments than in CON (p < 0.001). Structural carbohydrate fractions, including NDF, NDFom, ADF, cellulose, and lignin, increased significantly following fermentation, with the greatest increases generally observed in the WRF-inoculated treatments (FPC, FTV, and FPCTV) (p < 0.05).

**Table 3:** Effects of single and consortium WRF fermentation on the nutrient composition of mixed oil palm by-product substrate.

**Nutrient component (% DM)**	**CON**	**FCON**	**FPC**	**FTV**	**FPCTV**	**p-value**
Dry matter	41.49 ± 0.22ᵃ	33.32 ± 0.40ᵇ	33.13 ± 0.64ᵇᶜ	31.91 ± 0.25ᶜ	32.79 ± 0.34ᵇᶜ	<0.001
Ash	8.92 ± 0.15ᵇ	10.29 ± 0.07ᵃ	10.58 ± 0.23ᵃ	13.40 ± 1.50ᵃᵇ	13.57 ± 0.82ᵃ	<0.001
Ether extract	2.50 ± 0.09ᵃ	0.55 ± 0.05ᵇᶜ	0.79 ± 0.14ᵇ	0.65 ± 0.11ᵇᶜ	0.37 ± 0.10ᶜ	<0.001
Crude protein	10.17 ± 0.48ᵇ	12.58 ± 0.35ᵃ	12.69 ± 0.33ᵃ	13.71 ± 0.32ᵃ	13.49 ± 0.49ᵃ	<0.001
NDF	70.83 ± 0.44ᶜ	75.23 ± 0.38ᵃ	72.52 ± 0.56ᵇ	72.47 ± 0.50ᵇ	72.71 ± 0.41ᵇ	<0.001
NDFom	61.48 ± 0.30ᶜ	67.54 ± 0.32ᵃ	65.14 ± 0.58ᵇ	65.25 ± 0.89ᵇ	63.32 ± 1.10ᵇᶜ	<0.001
ADF	51.07 ± 0.85ᵇ	58.39 ± 0.21ᵃ	58.41 ± 0.33ᵃ	58.96 ± 0.48ᵃ	59.75 ± 0.93ᵃ	<0.001
Cellulose	38.27 ± 0.73ᵇ	41.54 ± 0.61ᵃ	41.46 ± 0.34ᵃ	41.02 ± 0.38ᵃ	41.44 ± 1.22ᵃ	0.021
Hemicellulose	19.76 ± 0.73ᵃ	16.84 ± 0.56ᵇ	14.12 ± 0.45ᶜ	13.51 ± 0.24ᶜ	12.96 ± 0.79ᶜ	<0.001
Lignin	12.80 ± 0.51ᵇ	16.85 ± 0.31ᵃ	16.95 ± 0.26ᵃ	17.95 ± 0.88ᵃ	18.31 ± 0.45ᵃ	<0.001
NFC	16.90 ± 0.58ᵃ	9.04 ± 0.24ᵇ	10.80 ± 0.69ᵇ	6.98 ± 1.37ᵇ	9.24 ± 1.42ᵇ	<0.001
Gross energy (MJ/kg DM)	18.06 ± 0.03ᵇ	18.81 ± 0.05ᵃ	18.80 ± 0.05ᵃ	19.26 ± 0.32ᵃ	18.67 ± 0.26ᵃ	0.005

DM = Dry matter, CON = Unfermented control, FCON = Fermentation control without fungal inoculation, FPC = Fermentation with *P. chrysosporium* InaCC F206, FTV = Fermentation with *T. versicolor* InaCC F200, FPCTV = Consortium of *P. chrysosporium* InaCC F206 and *T. versicolor* InaCC F200, NDF = Neutral detergent fiber, NDFom = Neutral detergent fiber corrected for residual ash, ADF = Acid detergent fiber, NFC = Non-fiber carbohydrates (calculated as 100 − [crude protein + ether extract + ash + NDFom] on a DM basis). Values within the same row with different superscript letters differ significantly (p < 0.05) according to Duncan's multiple range test. Data are presented as mean ± SE (n = 5).

### *In vitro* rumen fermentation characteristics

WRF fermentation did not significantly affect ruminal pH or NH₃-N concentration (p > 0.05) (Table 4). However, total VFA concentration was significantly lower in all fermented treatments than in CON (p = 0.031). Fermentation also significantly altered the molar proportions of individual VFAs. Compared with CON, the proportions of acetate, iso-butyrate, and iso-valerate increased significantly, whereas the proportion of butyrate decreased in all fermented treatments (p < 0.001). Propionate proportion was significantly increased only in FPC (p = 0.015), whereas valerate differed only slightly among treatments (p = 0.008). Consequently, the A:P ratio differed significantly among treatments (p = 0.043). Estimated CH₄ production and hydrogen-adjusted CH₄ production were significantly reduced in FCON, FPC, FTV, and FPCTV compared with CON (p < 0.05).

### *In vitro* gas production, gas kinetics, and digestibility

**Figure 1:**
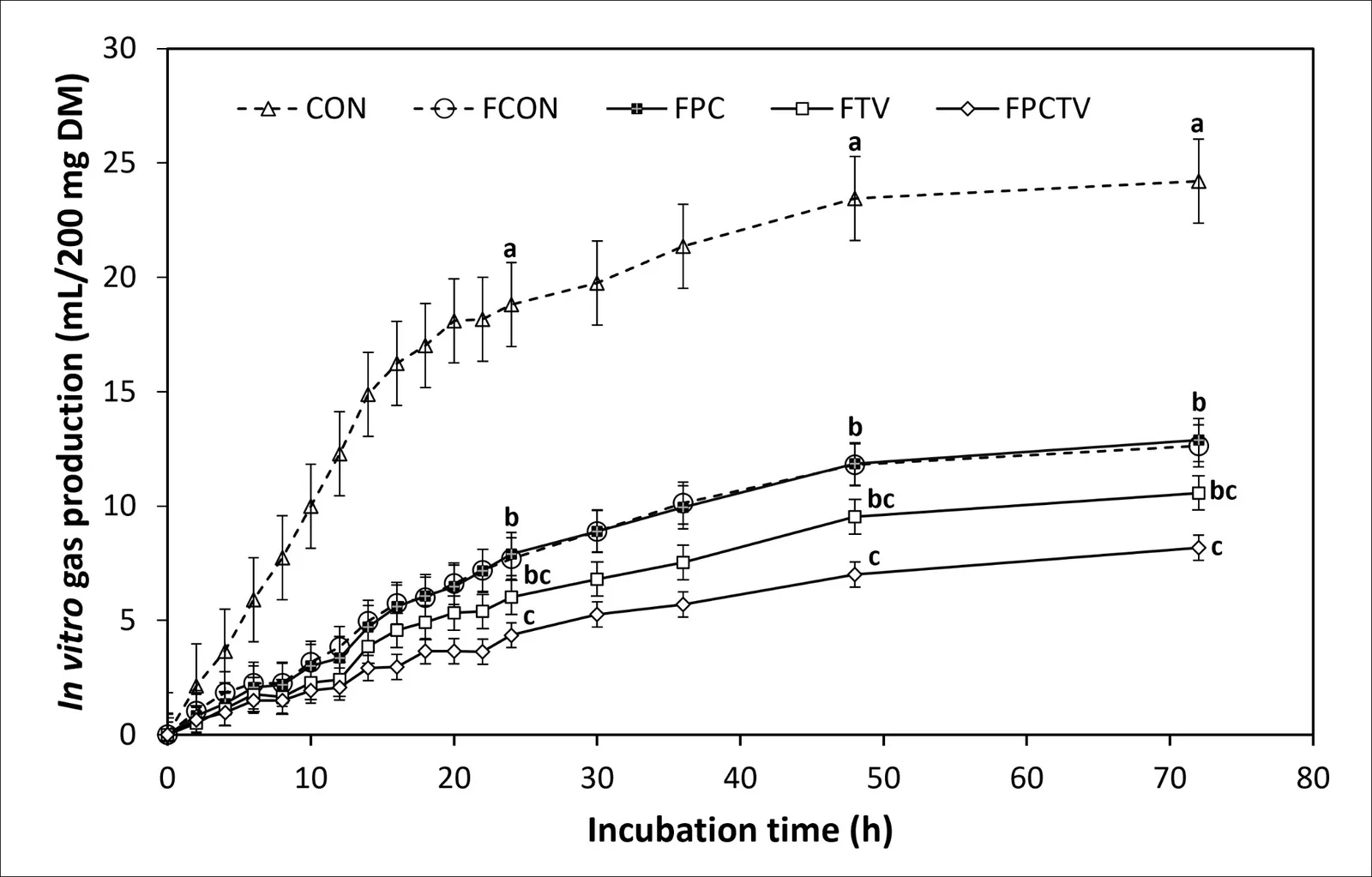
Cumulative *in vitro* gas production of mixed oil palm by-product substrate incubated under different fermentation treatments. CON = Unfermented control, FCON = Fermentation control without fungal inoculation, FPC = Fermentation with *P. chrysosporium* InaCC F206, FTV = Fermentation with *T. versicolor* InaCC F200, FPCTV = Consortium of *P. chrysosporium* InaCC F206 and *T. versicolor* InaCC F200, DM = Dry matter. Error bars represent the mean ± SE of five biological replicates (n = 5). Different superscript letters at 24, 48, and 72 h indicate significant differences among treatments (p < 0.05). Overlapping curves (FCON and FPC) share the same superscript letter (b).

Cumulative gas production increased progressively with incubation time in all treatments (Figure 1). However, cumulative gas production was consistently lower in FCON, FPC, FTV, and FPCTV than in CON throughout the incubation period (p < 0.01). Gas production from the immediate soluble fraction (a) was significantly greater in all fermented treatments than in CON (p = 0.002). In contrast, gas production from the insoluble fraction (b), total potential gas production (a + b), and the fractional rate constant of gas production from the insoluble fraction (c) were significantly lower in the fermented treatments than in CON (p < 0.01). Detailed cumulative gas production and kinetic parameters are presented in Table 5.

**Table 4:** Effects of single- and consortium-based WRF fermentation on in vitro rumen fermentation characteristics of a mixed oil palm by-product substrate.

**Variable**	**CON**	**FCON**	**FPC**	**FTV**	**FPCTV**	**p-value**
pH	7.05 ± 0.03	7.03 ± 0.04	6.99 ± 0.06	7.08 ± 0.04	7.06 ± 0.03	0.577
NH₃-N (mM)	1.55 ± 0.18	1.60 ± 0.05	1.40 ± 0.06	1.71 ± 0.20	1.41 ± 0.10	0.201
Total VFA (mM)	95.19 ± 7.59ᵃ	77.95 ± 8.10ᵃᵇ	71.05 ± 7.51ᵇ	64.07 ± 3.30ᵇ	68.79 ± 5.62ᵇ	0.031
Acetate (%)	61.17 ± 0.06ᵇ	61.65 ± 0.13ᵃ	61.56 ± 0.10ᵃ	61.63 ± 0.09ᵃ	61.64 ± 0.11ᵃ	0.016
Propionate (%)	20.50 ± 0.09ᵇ	20.48 ± 0.08ᵇ	20.76 ± 0.03ᵃ	20.51 ± 0.05ᵇ	20.36 ± 0.09ᵇ	0.015
Iso-butyrate (%)	2.52 ± 0.03ᶜ	2.93 ± 0.04ᵇ	2.85 ± 0.06ᵇ	2.94 ± 0.04ᵃᵇ	3.05 ± 0.02ᵃ	<0.001
Butyrate (%)	8.21 ± 0.04ᵃ	6.95 ± 0.10ᵇ	6.89 ± 0.08ᵇ	6.85 ± 0.04ᵇ	6.77 ± 0.04ᵇ	<0.001
Iso-valerate (%)	5.12 ± 0.04ᶜ	5.71 ± 0.05ᵃᵇ	5.61 ± 0.07ᵇ	5.71 ± 0.06ᵃᵇ	5.86 ± 0.04ᵃ	<0.001
Valerate (%)	1.87 ± 0.03	1.87 ± 0.02ᵃᵇ	1.88 ± 0.01ᵇ	1.91 ± 0.01ᵃᵇ	1.94 ± 0.01ᵃ	0.008
A:P	2.99 ± 0.01ᵃᵇ	3.01 ± 0.02ᵃ	2.97 ± 0.01ᵇ	3.00 ± 0.01ᵃᵇ	3.03 ± 0.02ᵃ	0.043
CH₄ (mmol/L)	23.96 ± 1.89ᵃ	19.41 ± 2.05ᵃᵇ	17.59 ± 1.87ᵇ	15.91 ± 0.81ᵇ	17.09 ± 1.38ᵇ	0.023
Adjusted CH₄ (mmol/L)	20.21 ± 1.60ᵃ	16.25 ± 1.73ᵃᵇ	14.63 ± 1.68ᵇ	13.31 ± 0.68ᵇ	14.27 ± 1.16ᵇ	0.022

CON = Unfermented control, FCON = Fermentation control without fungal inoculation, FPC = Fermentation with *P. chrysosporium* InaCC F206, FTV = Fermentation with *T. versicolor* InaCC F200, FPCTV = Consortium of *P. chrysosporium* InaCC F206 and *T. versicolor* InaCC F200, NH₃-N = Ammonia nitrogen, VFA = Volatile fatty acids, A:P = Acetate-to-propionate ratio, CH₄ = Methane, Adjusted CH₄ = Methane production adjusted for hydrogen recovery according to Jayanegara *et al.* [31]. Values within the same row with different superscript letters differ significantly (p < 0.05) according to Duncan's multiple range test. Data are presented as mean ± SE (n = 5).

**Table 5:** Effects of single and consortium WRF fermentation on in vitro gas production and gas kinetics of mixed oil palm by-product substrate.

**Variable**	**CON**	**FCON**	**FPC**	**FTV**	**FPCTV**	**p-value**
Net gas production (mL/200 mg DM)						
2 h	2.14 ± 0.29ᵃ	1.00 ± 0.16ᵇ	0.84 ± 0.43ᵇ	0.50 ± 0.00ᵇ	0.64 ± 0.21ᵇ	0.002
4 h	3.66 ± 0.39ᵃ	1.84 ± 0.27ᵇ	1.34 ± 0.43ᵇ	1.10 ± 0.10ᵇ	0.96 ± 0.41ᵇ	0.003
8 h	7.76 ± 0.37ᵃ	2.28 ± 0.11ᵇ	2.20 ± 0.56ᵇ	1.66 ± 0.21ᵇ	1.50 ± 0.45ᵇ	<0.001
12 h	12.28 ± 0.47ᵃ	3.84 ± 0.30ᵇ	3.34 ± 0.82ᵇᶜ	2.44 ± 0.37ᵇᶜ	2.08 ± 0.63ᶜ	<0.001
24 h	18.82 ± 0.55ᵃ	7.70 ± 0.44ᵇ	7.92 ± 1.43ᵇ	6.00 ± 0.64ᵇᶜ	4.34 ± 0.67ᶜ	<0.001
48 h	23.46 ± 0.64ᵃ	11.78 ± 0.69ᵇ	11.84 ± 1.65ᵇ	9.52 ± 0.83ᵇᶜ	7.02 ± 1.01ᶜ	<0.001
72 h	24.18 ± 0.61ᵃ	12.64 ± 0.64ᵇ	12.88 ± 1.50ᵇ	10.58 ± 0.83ᵇᶜ	8.18 ± 1.06ᶜ	<0.001
Gas production parameters						
a (mL)	−2.09 ± 0.26ᵇ	−0.39 ± 0.33ᵃ	−0.72 ± 0.37ᵃ	−0.47 ± 0.17ᵃ	0.02 ± 0.39ᵃ	0.002
b (mL)	24.65 ± 0.49ᵃ	14.86 ± 0.77ᵇ	15.58 ± 1.30ᵇ	12.85 ± 0.91ᵇᶜ	10.29 ± 1.01ᶜ	<0.001
c (mL/h)	0.068 ± 0.001ᵃ	0.031 ± 0.002ᵇ	0.029 ± 0.004ᵇ	0.028 ± 0.003ᵇᶜ	0.020 ± 0.002ᶜ	<0.001
a + b (mL)	22.57 ± 0.61ᵃ	14.47 ± 0.47ᵇ	14.86 ± 1.25ᵇ	12.38 ± 0.79ᵇᶜ	10.32 ± 0.97ᶜ	<0.001
R²	0.99	0.98	0.99	0.98	0.96	—

DM = Dry matter, CON = Unfermented control, FCON = Fermentation control without fungal inoculation, FPC = Fermentation with *P. chrysosporium* InaCC F206, FTV = Fermentation with *T. versicolor* InaCC F200, FPCTV = Consortium of *P. chrysosporium* InaCC F206 and *T. versicolor* InaCC F200, a = Gas production from the immediate soluble fraction, b = Gas production from the insoluble fraction, c = Fractional rate constant of gas production from the insoluble fraction, a + b = Total potential gas production, R² = Coefficient of determination (goodness-of-fit). Values within the same row with different superscript letters differ significantly (p < 0.05) according to Duncan's multiple range test. Data are presented as mean ± SE (n = 5).

IVDMD and IVOMD were significantly affected by treatment (Table 6). The highest digestibility values were observed in CON, whereas FPCTV exhibited the lowest values (p < 0.001). ME followed a similar trend, with all fermented treatments (FCON, FPC, FTV, and FPCTV) exhibiting significantly lower ME than CON (p < 0.001).

**Table 6:** Effects of single and consortium WRF fermentation on in vitro digestibility and metabolizable energy of mixed oil palm by-product substrate.

**Variable**	**CON**	**FCON**	**FPC**	**FTV**	**FPCTV**	**p-value**
IVDMD (%)	57.43 ± 1.40ᵃ	34.19 ± 0.68ᵇ	36.39 ± 3.05ᵇ	34.99 ± 1.95ᵇ	27.35 ± 1.02ᶜ	<0.001
IVOMD (%)	43.37 ± 0.81ᵃ	36.32 ± 0.48ᵇ	36.75 ± 0.97ᵇ	37.67 ± 0.99ᵇ	36.25 ± 0.89ᵇ	<0.001
ME (MJ/kg DM)	5.26 ± 0.13ᵃ	3.37 ± 0.09ᵇ	3.46 ± 0.22ᵇ	3.23 ± 0.13ᵇᶜ	2.90 ± 0.10ᶜ	<0.001

CON = Unfermented control, FCON = Fermentation control without fungal inoculation, FPC = Fermentation with *P. chrysosporium* InaCC F206, FTV = Fermentation with *T. versicolor* InaCC F200, FPCTV = Consortium of *P. chrysosporium* InaCC F206 and *T. versicolor* InaCC F200, IVDMD = *In vitro* dry matter digestibility, IVOMD = *In vitro* organic matter digestibility, ME = Metabolizable energy estimated using the equations of Menke and Steingass [26], DM = Dry matter. Values within the same row with different superscript letters differ significantly (p < 0.05) according to Duncan's multiple range test. Data are presented as mean ± SE (n = 5).

### Rumen microbial population

WRF fermentation significantly affected the total bacterial population (Table 7), with the highest abundance observed in FCON (p = 0.012). In contrast, the populations of *F. succinogenes* and *Ruminococcus albus* were not significantly affected by the fermentation treatments (p > 0.05).

**Table 7:** Effects of single- and consortium-based WRF fermentation on rumen microbial populations in a mixed oil palm by-product substrate.

**Microbe (Log₁₀ copy number/mL)**	**CON**	**FCON**	**FPC**	**FTV**	**FPCTV**	**p-value**
Total bacteria	12.40 ± 0.07ᵃᵇ	12.62 ± 0.09ᵃ	12.22 ± 0.03ᵇ	12.31 ± 0.10ᵇ	12.47 ± 0.03ᵃᵇ	0.012
*Fibrobacter* * succinogenes*	6.08 ± 0.34	5.66 ± 0.74	5.52 ± 0.81	5.31 ± 0.85	3.69 ± 0.34	0.153
*Ruminococcus* * albus*	9.78 ± 0.09	9.53 ± 0.39	9.36 ± 0.50	9.79 ± 0.17	9.69 ± 0.24	0.850

CON = Unfermented control, FCON = Fermentation control without fungal inoculation, FPC = Fermentation with *P. chrysosporium* InaCC F206, FTV = Fermentation with *T. versicolor* InaCC F200, FPCTV = Consortium of *P. chrysosporium* InaCC F206 and *T. versicolor* InaCC F200. Values within the same row with different superscript letters differ significantly (p < 0.05) according to Duncan's multiple range test. Data are presented as mean ± SE (n = 4).

### Correlation analysis between chemical composition and *in vitro* fermentation parameters

**Figure 2:**
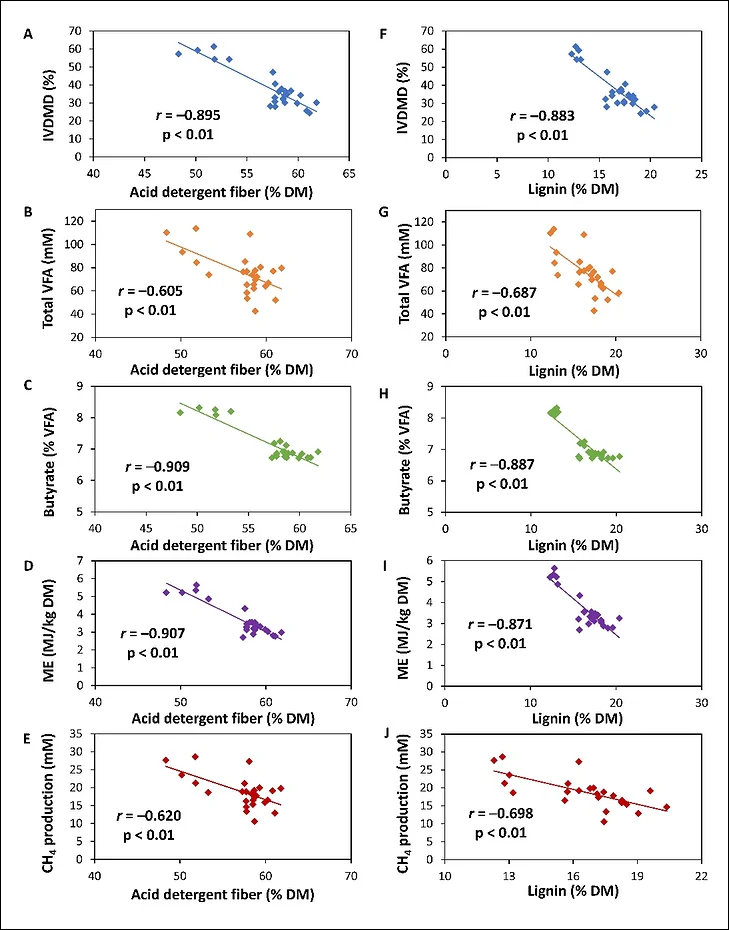
Correlations between fiber fractions of the substrates and *in vitro* rumen fermentation parameters. Relationships between ADF (% DM) and (A) IVDMD (%), (B) total VFA (mM), (C) butyrate (% of total VFA), (D) ME (MJ/kg DM), and (E) CH₄ production (mM). Relationships between lignin concentration (% DM) and (F) IVDMD (%), (G) total VFA (mM), (H) butyrate (% of total VFA), (I) ME (MJ/kg DM), and (J) CH₄ production (mM). Pearson's correlation coefficients (r) and corresponding p-values are presented in each panel. ADF = Acid detergent fiber, CH₄ = Methane, DM = Dry matter, IVDMD = *In vitro* dry matter digestibility, ME = Metabolizable energy, VFA = Volatile fatty acids.

IVDMD, IVOMD, and ME exhibited similar correlation patterns with the chemical composition of the substrate (Table 8). These variables were negatively correlated with CP, ADF, and lignin concentration (p < 0.01) and positively correlated with EE (p < 0.01). NDF showed moderate negative correlations with IVDMD, IVOMD, and ME (p < 0.05). Total VFA concentration and CH₄ production were negatively correlated with CP (p < 0.05) and ADF and lignin concentrations (p < 0.01), but positively correlated with EE. Among the fiber fractions, ADF and lignin concentration consistently exhibited strong negative correlations with IVDMD, total VFA, butyrate proportion, ME, and CH₄ production (p < 0.01) (Figure 2). In contrast, NH₃-N concentration was not significantly correlated with any of the measured chemical composition variables (p > 0.05).

**Table 8:** Correlation coefficients between chemical composition and in vitro rumen fermentation parameters of mixed oil palm by-product substrate.

**Variable**	**CP**	**NDF**	**ADF**	**Lignin**	**EE**	**Ash**
IVDMD	−0.773**	−0.499*	−0.895**	−0.883**	0.938**	−0.606**
IVOMD	−0.571**	−0.591**	−0.810**	−0.743**	0.881**	−0.211
Total VFA	−0.444*	−0.139	−0.605**	−0.687**	0.608**	−0.399*
Acetate	0.476*	0.404*	0.550**	0.573**	−0.576**	0.323*
Propionate	0.029	−0.027	−0.059	−0.180	0.070	−0.320
Butyrate	−0.805**	−0.497*	−0.909**	−0.887**	0.947**	−0.552**
NH₃-N	−0.195	0.180	0.046	0.054	0.050	−0.009
CH₄	−0.460*	−0.148	−0.620**	−0.698**	0.624**	−0.406*
ME	−0.743**	−0.520**	−0.907**	−0.871**	0.973**	−0.591**

CP = Crude protein, NDF = Neutral detergent fiber, ADF = Acid detergent fiber, EE = Ether extract, IVDMD = *In vitro* dry matter digestibility, IVOMD = *In vitro* organic matter digestibility, VFA = Volatile fatty acids, NH₃-N = Ammonia nitrogen, CH₄ = Methane production, ME = Metabolizable energy estimated using the equations of Menke and Steingass [26]. *p < 0.05, **p < 0.01.

## DISCUSSION

### Changes in nutrient composition

Current knowledge of WRF pretreatment of oil palm by-products is derived primarily from studies using individual substrates, particularly OPF and PKC treated with *P. chrysosporium* [16, 18]. In contrast, the present study evaluated the fermentation of a mixed oil palm by-product substrate comprising OPF, PKC, and OPDC using single cultures and a consortium of *P. chrysosporium* and *T. versicolor*. This approach provides a more practical assessment of WRF performance under heterogeneous substrate conditions that better represent oil palm by-products used as ruminant feed resources.

WRF fermentation reduced the DM content of the mixed oil palm by-product substrate. This reduction is consistent with the general mechanism of WRF degradation, in which polysaccharides such as cellulose and hemicellulose are utilized as carbon and energy sources, resulting in DM loss and the release of CO₂ and H₂O [11, 20]. Previous studies have shown that the extent of DM loss is influenced by fungal growth, metabolic activity, and substrate characteristics [37]. However, the comparable reductions in DM observed between fungal-inoculated treatments and spontaneous fermentation indicate that DM loss was attributable not solely to WRF activity but also to the overall fermentation process.

Although not statistically significant, the FTV treatment showed the greatest numerical reduction in DM (23.09%) compared with the other fermented treatments (19.69%–20.97%). This differs from observations in single-substrate studies, in which *P. chrysosporium* caused less DM loss than *T. versicolor* [38], suggesting that substrate composition influences fungal degradation responses. Mixed oil palm by-products contain heterogeneous components, including residual lipids and mannan derived from PKC and OPDC [39], which may affect fungal metabolism and colonization. Unlike individual substrates with relatively uniform composition, the mixed substrate provides multiple nutrient sources that may alter fungal colonization patterns and degradation behavior. Under these conditions, *T. versicolor*, a non-selective ligninolytic fungus, may degrade a broader range of fiber components [40]. In contrast, the FPCTV treatment did not produce greater DM loss than the single-culture treatments. A similar observation has been reported for white tea residue, indicating that WRF consortia do not necessarily increase the overall intensity of substrate degradation [20].

The reductions in EE and NFC observed across all fermented treatments indicate that readily fermentable, non-structural components were preferentially utilized during the initial stages of fungal colonization, thereby increasing the relative proportion of structural carbohydrates. Similar responses have been reported in other lignocellulosic substrates, where depletion of soluble fractions results in an apparent increase in the abundance of fiber components [20, 40]. Unlike studies using individual substrates that reported strain-dependent responses [38], the mixed substrate evaluated in the present study showed relatively consistent reductions in EE among fungal treatments, suggesting that interactions between substrate composition and fungal metabolism may have moderated strain-specific effects.

The increase in CP observed after fermentation is likely explained by two complementary mechanisms. First, fungal biomass contributes additional nitrogen to the substrate [37, 41]. Chitin, a major structural component of fungal cell walls, is a nitrogen-containing polysaccharide that contributes to this increase [42]. Second, the degradation of non-structural carbohydrates and the accompanying reduction in DM increase the relative concentration of CP [37]. Similar increases in CP have been reported for OPF and PKC fermented with *P. chrysosporium* [16, 18], PKC fermented with *Aspergillus niger* [17], and OPDC fermented with *Rhizopus oligosporus* [43].

The increases in ADF and lignin concentrations primarily reflect proportional changes in cell wall composition following the preferential degradation of more readily fermentable fractions. During SSF, WRF initially consume soluble carbohydrates, hemicellulose, and other non-structural components as immediate energy sources, resulting in a reduction in substrate DM. Consequently, the more recalcitrant lignin fraction becomes proportionally concentrated within the remaining biomass. Thus, lignin appears to increase on a DM basis even though its absolute amount may remain unchanged or decrease only slightly. Although this apparent increase may partly reflect methodological limitations associated with detergent fiber analysis following fungal treatment [44], the concurrent reduction in hemicellulose supports preferential carbohydrate degradation as the principal explanation for the higher relative lignin concentration. Similar responses have been widely reported for lignocellulosic biomass, in which carbohydrate degradation initially exceeds delignification [45].

A comparable pattern has also been reported in OPF fermented with *P. chrysosporium* and *T. versicolor*, including spontaneous fermentation [23]. Hemicellulose, owing to its amorphous structure, is generally more susceptible to hydrolysis than crystalline cellulose [46], consistent with the significant reduction in hemicellulose observed in the present study. *P. chrysosporium* is considered a relatively selective WRF capable of degrading lignin and hemicellulose through the production of ligninolytic enzymes and glycoside hydrolases [47–49]. In contrast, *T. versicolor* exhibits a less selective degradation pattern and can decompose multiple lignocellulosic components, as reflected by greater reducing sugar production [23, 40]. However, these characteristics have been described mainly in studies using individual substrates, and their degradation behavior may differ when both fungi are applied to a heterogeneous mixed oil palm by-product substrate containing diverse lignocellulosic components.

The present study further demonstrated that the consortium treatment (FPCTV) produced greater hemicellulose degradation without improving selective delignification. Although synergistic interactions between *P. chrysosporium* and *T. versicolor* have been reported previously [14], competitive interactions may also occur during co-cultivation. Faster colonization by *P. chrysosporium* may suppress the establishment and activity of *T. versicolor*, as observed in agar-based assays [50]. Such competition may explain the greater degradation of hemicellulose without a corresponding improvement in delignification efficiency.

Differences in incubation period may also contribute to discrepancies among published studies. The efficiency of lignocellulose degradation by WRF depends strongly on fermentation duration, with the optimal incubation period varying with fungal species, substrate characteristics, and fermentation conditions rather than on a fixed incubation time [51]. Consequently, incubation time should be optimized for each substrate to maximize delignification while minimizing nutrient losses. Variations in incubation period may partly explain the differing responses reported for *P. chrysosporium* and *T. versicolor* in previous studies [16, 18, 23, 38]. The 28-day incubation period used in the present study was selected based on previous investigations of lignocellulosic pretreatment using these fungi [21–23]. However, the SSF conditions were not specifically optimized for the mixed oil palm by-product substrate. Therefore, the limited improvements observed in the present study may reflect suboptimal fermentation conditions rather than the inherent ineffectiveness of WRF pretreatment. Further optimization of SSF conditions, including incubation period, inoculum composition, and culture conditions, is required to maximize the effectiveness of WRF pretreatment for mixed oil palm by-products.

### *In vitro* rumen fermentation characteristics and methane production

WRF fermentation did not alter rumen pH, with all treatments maintaining values within the physiological range required for optimal rumen microbial growth and activity [52]. This finding indicates that the differences observed in fermentation characteristics among treatments were not attributable to changes in ruminal pH.

NH₃-N concentrations remained unchanged despite the higher CP content of the fermented substrates, suggesting that a proportion of the additional protein was not readily degradable under the *in vitro* rumen conditions. Similar observations have been reported for PKC fermented with *A.* *niger* and mannanase and for OPF fermented with *P. chrysosporium* [17, 18], in which NH₃-N concentrations remained within the physiological range required for microbial protein synthesis [52]. The increased CP content in the present study most likely reflects the accumulation of fungal biomass, which may not be readily degraded by rumen microorganisms. Consequently, the synchrony between fermentable energy and degradable nitrogen may have been suboptimal, thereby limiting microbial growth and fermentation [53] and contributing to the lower VFA concentrations observed.

All substrates fermented with *P. chrysosporium*, *T. versicolor*, and their consortium produced lower total VFA concentrations than the unfermented control, consistent with the reductions in gas production and IVDMD. These findings suggest that limited degradation of structural carbohydrates reduced the availability of fermentable substrates for rumen microorganisms, as has been reported in previous studies [18, 54].

Rumen VFA production is strongly influenced by diet composition and the structure of the rumen microbial community [55]. Changes in substrate composition modify fermentation pathways because readily fermentable carbohydrates are metabolized more rapidly than structural carbohydrates. For example, replacing corn and soybean meal with fermented PKC reduced butyrate production [17]. Such changes in substrate composition influence rumen microbial metabolism and hydrogen (H₂) production, thereby altering VFA profiles [56, 57].

The decline in total VFA concentration observed in the present study reflects reduced overall fermentation intensity and, consequently, lower H₂ production. During acetate and butyrate formation, H₂ is generated and subsequently utilized by methanogenic archaea for CH₄ production [58]. Therefore, the reduced degradation of fermentable carbohydrates likely decreased H₂ availability, resulting in lower CH₄ production. This finding indicates that the reduction in CH₄ was primarily due to reduced fermentation intensity rather than improved ruminal fermentation efficiency, consistent with previous reports [56]. Although lower CH₄ emissions are generally considered environmentally beneficial, the concurrent reductions in VFA production and digestibility indicate that CH₄ mitigation occurred at the expense of nutrient utilization. Therefore, the reduced CH₄ production observed in the present study should not be interpreted as evidence of improved fermentation efficiency or an effective enteric CH₄ mitigation strategy. Further *in vivo* studies are needed to determine whether optimization of WRF pretreatment can simultaneously improve nutrient utilization, maintain animal performance, and reduce CH₄ emissions.

### *In vitro* gas production, gas kinetics, and digestibility

Contrary to the expected benefits of biological pretreatment, fermentation of mixed oil palm by-products with *P. chrysosporium*, *T. versicolor*, or their consortium reduced IVDMD, IVOMD, and ME. These findings indicate that WRF pretreatment did not improve nutrient digestibility or energy availability under the conditions used in the present study. The decline in digestibility is likely associated with changes in carbohydrate composition during fermentation. As discussed above, WRF fermentation reduced NFC and preferentially degraded hemicellulose, resulting in a proportional increase in lignin concentration. Lignin acts as a physical barrier that restricts the access of cellulolytic enzymes to structural carbohydrates, while its hydrophobic properties promote non-productive enzyme adsorption [59], thereby reducing microbial degradation and feed digestibility. Because lignocellulose consists of a complex matrix of lignin, cellulose, and hemicellulose linked through ester and ether bonds [46], preferential removal of carbohydrate fractions without sufficient delignification is unlikely to improve the accessibility of structural carbohydrates to rumen microorganisms.

These findings agree with previous reports showing that IVDMD and IVOMD decreased as the dietary inclusion of *P. chrysosporium*-fermented OPF replacing Napier grass increased [18]. Increased lignin concentration has been proposed to reduce the surface area available for microbial attachment, thereby limiting cellulolytic bacterial colonization and fiber degradation [11]. This mechanism is further supported by the lower values of the gas production parameters b, c, and a + b observed in the present study, indicating that WRF pretreatment did not substantially improve degradation of the structural carbohydrate fraction. Consequently, ruminal fermentation proceeded more slowly and exhibited lower overall fermentation potential, particularly in the FPCTV treatment. The reduced fraction of degradable substrate also explains the lower cumulative gas production, consistent with observations in *T. versicolor*-fermented *Brassica* straw [54].

Nevertheless, these results should not be interpreted as evidence that WRF pretreatment is inherently ineffective for mixed oil palm by-products. A recent meta-analysis of OPF pretreatment strategies demonstrated that biological pretreatment generally improves rumen digestibility and fermentability [5]. Therefore, the contrasting results obtained in the present study suggest that the effectiveness of WRF depends on substrate composition and fermentation conditions rather than on fungal pretreatment alone.

Mixed oil palm by-products have also been evaluated using alternative bioprocessing approaches. Yulistiani *et al.* [6] investigated isonitrogenous ensiled complete diets containing OPF, PKC, and OPDC and reported numerically greater gas production and digestibility than those observed in the present study. However, those parameters did not differ significantly from the control diet containing OPF and PKC without OPDC. Consequently, direct comparisons between the two studies should be interpreted cautionwith because due to substantial differences in bioprocessing methods, diet formulation, and experimental design.

### Rumen microbial populations

The higher total bacterial population observed in FCON than in the WRF-treated substrates suggests that fungal pretreatment influenced subsequent rumen bacterial growth. Although the fermented substrates were compositionally similar, microbial competition for available nutrients during fermentation may have reduced bacterial proliferation. In addition, the higher ADF and lignin concentrations in the WRF-treated substrates may have limited substrate accessibility for bacterial colonization and growth [11].

The absence of detectable protozoa in the present study may indicate that their abundance was below the detection limit of the qPCR assay. In goats fed PKC, reduced protozoal populations have been associated with increased bacterial abundance, possibly due to high concentrations of unsaturated fatty acids that exert toxic effects on rumen protozoa [60]. Similarly, diets containing OPF, PKC, or OPDC have been reported to suppress rumen protozoa in cattle, sheep, and goats while increasing total bacterial populations [60–64]. These observations are consistent with the ecological role of protozoa as major predators of rumen bacteria.

Fibrolytic bacteria, including *F. succinogenes*, *R. albus*, and *Ruminococcus* *flavefaciens*, play key roles in the degradation of structural carbohydrates [65]. Increasing dietary NDF generally stimulates the growth of cellulolytic bacteria [66]. In the present study, NDF increased significantly only in FCON, whereas no differences were observed among FPC, FTV, and FPCTV. However, this increase was not accompanied by changes in the populations of *F. succinogenes* or *R. albus*, both of which declined in the WRF-treated substrates. Although these differences were not statistically significant, the observed trend may indicate reduced fibrolytic activity following WRF pretreatment. This response may be associated with the relatively high lignin and ADF concentrations in the fermented substrates, which reduce microbial attachment and limit enzymatic access to cellulose, thereby decreasing fiber degradation [11]. Previous studies have also demonstrated that the abundance of dominant fibrolytic bacteria varies according to animal species and dietary composition [60, 66–68].

### Correlation between chemical composition and *in vitro* fermentation parameters

Correlation analysis identified fiber fractions, particularly ADF and lignin concentrations, as the principal factors associated with the rumen fermentation response to WRF-treated mixed oil palm by-products. The strong negative correlations between ADF and lignin and IVDMD, IVOMD, ME, total VFA, butyrate, and CH₄ production indicate that structural fiber composition remained the primary constraint on substrate degradation. Similar relationships have been reported previously [69].

In contrast, EE exhibited the opposite correlation pattern, indicating that substrates with lower lignin concentrations generally retained higher lipid contents and supported greater fermentability, digestibility, and energy value. Although CP increased following WRF treatment, its negative correlations with several fermentation variables indicate that the higher CP concentration did not necessarily improve the nutritional value of the substrate for rumen microorganisms. This apparent increase in CP most likely resulted from fungal biomass accumulation [37, 41], while the concurrent depletion of fermentable carbohydrate fractions reduced the energy available to support microbial fermentation. Collectively, these findings suggest that successful WRF pretreatment should preserve fermentable carbohydrate fractions while enhancing protein content to improve ruminal fermentation and nutrient utilization.

### Fungal consortium performance

The present study demonstrated that the consortium of *P. chrysosporium* and *T. versicolor* (FPCTV) did not outperform the respective single-culture treatments in improving the rumen fermentability and digestibility of mixed oil palm by-products. Previous studies have reported synergistic interactions among these fungi, primarily through enhanced production of lignocellulolytic enzymes on selected lignocellulosic substrates [14]. However, the expression and activity of these enzymes are highly dependent on substrate composition and fermentation conditions. Consequently, the synergistic interactions reported previously did not translate into improved rumen fermentation or digestibility under the conditions of the present study.

The limited effectiveness of the consortium may be explained by interspecific fungal interactions during fermentation. Competitive or antagonistic interactions may have restricted the establishment of complementary metabolic activities. Differences in substrate colonization may also have contributed, as the relatively rapid growth of *P. chrysosporium* on agar media [50] could provide a competitive advantage during the early stages of fermentation, thereby limiting the establishment of *T. versicolor*. Furthermore, the two fungi employ different lignocellulose degradation strategies. *T. versicolor* is a non-selective ligninolytic fungus capable of degrading multiple cell wall components [40], whereas *P. chrysosporium* exhibits relatively greater selectivity toward lignin and hemicellulose degradation [49]. These contrasting degradation strategies may have limited the development of complementary interactions when both fungi were cultured on a heterogeneous mixed oil palm by-product substrate. Consequently, the consortium failed to provide greater benefits than the individual fungal treatments.

In addition to fungal interactions, the incubation period may have contributed to the limited response observed for the consortium. As discussed previously, incubation time is a critical determinant of WRF pretreatment efficiency, and the optimal duration depends on fungal species, substrate characteristics, and fermentation conditions rather than a fixed incubation period [51]. Therefore, the 28-day incubation period used in the present study may not have been optimal for consortium fermentation, potentially limiting the expected synergistic interactions.

Previous investigations of fungal pretreatment have focused predominantly on individual lignocellulosic substrates, whereas comparatively few studies have evaluated mixed substrates using both single and consortium fungal cultures. To place the present findings into context, Table 9[16–18, 20] summarizes representative studies on fungal pretreatment and compares their substrates, fungal treatments, and major outcomes with those obtained in the current study.

**Table 9:** Comparison of the present study with previous studies on fungal pretreatment of lignocellulosic substrates.

**Reference**	**Substrate**	**Pretreatment**	**Reported outcomes**	**Comparison/implication**
[16]	Single (PKC)	*P. * *chrysosporium*	Increased CP and reduced lignin	Similar increase in CP, but the present study observed an increase rather than a reduction in relative lignin concentration.
[17]	Single (PKC)	*A. niger* + mannanase	Increased CP and digestibility; reduced NH₃-N and butyrate	Increase in CP was consistent with the present study; however, digestibility responses differed.
[18]	Single (OPF)	*P. * *chrysosporium*	Reduced digestibility and rumen fermentation	Similar decline in rumen fermentability was observed in a single OPF substrate.
[20]	Single (white tea residue)	*P. chrysosporium*, *P. ostreatus*, consortium	Increased CP, altered VFA production, and single-culture outperformed the consortium	Consistent with the present finding that the consortium did not outperform single cultures.
Present study	Mixed (OPF, PKC, and OPDC)	*P. chrysosporium*, *T. versicolor*, consortium	Increased CP and relative lignin concentration; reduced rumen fermentability, digestibility, gas production, and CH₄ production; consortium showed no advantage over single cultures	Extends previous WRF studies by evaluating both single and consortium fungal pretreatments of mixed oil palm by-products under conditions that more closely represent practical ruminant feeding systems.

OPF = Oil palm fronds, PKC = Palm kernel cake, OPDC = Oil palm decanter cake, CP = Crude protein, NH₃-N = Ammonia nitrogen, VFA = Volatile fatty acids, CH₄ = Methane production, WRF = White-rot fungi, *P. chrysosporium* = *Phanerochaete* *chrysosporium*, *A. niger* = *Aspergillus niger*, *P. ostreatus* = *Pleurotus ostreatus*, *T. versicolor* = *Trametes versicolor*.

### Study limitations and future perspectives

Several limitations of this study should be acknowledged. First, the experiment was conducted *in vitro*, which cannot fully replicate the complexity of rumen physiology, microbial adaptation, host–microbiome interactions, or animal responses observed *in vivo*. Consequently, the observed effects on nutrient utilization and fermentation should be interpreted with caution when extrapolating to practical feeding systems.

Second, although this study established the effects of WRF pretreatment on nutrient composition, rumen fermentation, digestibility, methane production, and microbial populations, the underlying mechanisms responsible for these responses were not directly investigated. In particular, ligninolytic enzyme production, enzyme kinetics, and structural modifications of the lignocellulosic matrix were not characterized. Future studies should integrate direct measurements of ligninolytic and cellulolytic enzyme activities with structural characterization techniques, such as Fourier-transform infrared spectroscopy, scanning electron microscopy, and other complementary analytical approaches, to better elucidate the mechanisms governing fungal degradation of mixed oil palm by-products.

Finally, the study was conducted under laboratory-scale SSF conditions using a single fungal inoculum ratio and a fixed 28-day incubation period. As discussed above, the effectiveness of WRF pretreatment depends heavily on fungal species, substrate composition, inoculum ratio, incubation period, and fermentation conditions. Therefore, further optimization of these parameters is required before practical application. In addition, future studies should evaluate the scalability, economic feasibility, and *in vivo* feeding performance of optimized WRF pretreatment systems to determine their suitability for sustainable ruminant production.

## CONCLUSION

This study demonstrated that SSF of mixed oil palm by-products using *P. chrysosporium*, *T. versicolor*, and their consortium substantially modified substrate chemical composition but did not improve ruminal fermentation characteristics under the conditions evaluated. Fungal pretreatment increased CP while reducing EE, NFC, and hemicellulose, resulting in proportional increases in ADF and lignin concentrations. These compositional changes were accompanied by lower total gas production, total VFA, butyrate, CH₄ production, IVDMD, IVOMD, and ME, whereas ruminal pH and NH₃-N remained unaffected. In addition, the fungal consortium did not outperform the single-fungus treatments, indicating that co-cultivation under the applied fermentation conditions did not yield synergistic effects that improved substrate utilization. Correlation analysis further identified ADF and lignin as the principal factors negatively influencing digestibility, fermentation efficiency, and energy availability.

From a practical perspective, the findings demonstrate that increasing CP through WRF pretreatment alone does not necessarily translate into improved nutritional value for ruminants. Effective bioprocessing of mixed oil palm by-products should simultaneously preserve fermentable carbohydrate fractions while selectively degrading lignin to enhance rumen microbial utilization. These results provide valuable guidance for developing biological pretreatment strategies for agro-industrial residues intended for ruminant feeding.

A major strength of this study is its comprehensive evaluation of chemical composition, *in vitro* rumen fermentation, gas kinetics, CH₄ production, digestibility, rumen microbial populations, and correlation analysis using a mixed oil palm by-product substrate that more closely represents practical feeding conditions than studies based on individual by-products. Furthermore, the direct comparison of single-fungus and consortium treatments provides new evidence that fungal co-cultivation does not inherently confer superior performance and that synergistic interactions are highly dependent on substrate characteristics and fermentation conditions.

Overall, this study demonstrates that the effectiveness of WRF pretreatment is highly substrate- and process-dependent. Although the fermentation conditions evaluated were insufficient to improve the nutritional value of mixed oil palm by-products, the findings provide an important foundation for refining fungal bioprocessing strategies and advancing the sustainable conversion of abundant oil palm residues into higher-value ruminant feed resources.

## DATA AVAILABILITY

The supplementary data can be made available from the corresponding author upon request.

## GENERATIVE AI DECLARATION

The authors declare that generative artificial intelligence (AI) tools were used solely to improve language, grammar, and readability during manuscript preparation. All scientific content, data analysis, interpretation of results, and conclusions were developed and verified by the authors. The authors take full responsibility for the accuracy, integrity, and originality of the work presented, and no AI tool was listed as an author.

## AUTHORS’ CONTRIBUTIONS

FAF and SS: Conceived and designed the study. FAF: Conducted the experimental work, performed laboratory analyses and data analysis, drafted the manuscript, and revised the manuscript. AJ, SW, AAS, and SS: Supervised the study, provided technical and methodological guidance, and critically reviewed the manuscript. All authors have read and approved the final version of the manuscript.
